# Therapeutic strategies for retention of cranioplasty titanium mesh after mesh exposure

**DOI:** 10.1007/s00701-022-05365-w

**Published:** 2022-10-10

**Authors:** Yao-Hua Zhao, Yu-Hong Feng, Hai-Tao Deng, Wei-Qi Huang, Li-Hong Xu, Xian-Ping Meng, Xu-Gang Xie

**Affiliations:** 1 Department of Burn and Plastic Surgery, Jiangyin Hospital Affiliated to School of Medicine Southeast University, Jiangyin, Jiangsu China; 2 Department of Nursing, Jiangyin Hospital Affiliated to School of Medicine Southeast University, Jiangyin, Jiangsu China; 3 Department of Radiology, Jiangyin Hospital Affiliated to School of Medicine Southeast University, Jiangyin, Jiangsu China

**Keywords:** Skull, Plastic surgery, Negative pressure wound therapy, Titanium, Prosthesis retention

## Abstract

**Background:**

Titanium mesh exposure after cranioplasty is a possible complication and is usually managed by mesh removal and flap transfer, but the advantages of the rigid prosthesis are then lost. This study aimed to present our experience with negative pressure wound therapy combined with soft tissue dilation for retaining the titanium mesh in patients with mesh exposure after cranioplasty.

**Methods:**

This retrospective study included patients treated between 01/2016 and 05/2019 at the Jiangyin Hospital Affiliated to Southeast University School of Medicine. The wound was cleaned, and a cystic space was created for the tissue dilator, which was used with a self-designed negative pressure dressing. After the target dilation was achieved, the repair was conducted while retaining the titanium mesh.

**Results:**

Eight patients were included (seven males and one female; 53.6 ± 8.8 (range, 43–65) years of age). The exposed mesh area ranged from 1 × 1 to 4 × 5.5 cm. The thinning scalp area around the exposed mesh ranged from 3.6 × 3.8 to 4 × 5.5 cm. Five patients had positive wound cultures and received sensitive antibiotics. The dilator embedding time was 20–28 days. The time of negative pressure wound therapy was 25–33 days. The hospital stay was 30–41 days. Primary wound healing was achieved in all eight patients. There were no signs of recurrence after 6–18 months of follow-up. The cranial CT scans were unremarkable.

**Conclusions:**

Negative pressure wound therapy combined with soft tissue dilation for exposed titanium mesh after cranioplasty might help retain the titanium mesh.

## Introduction

Cranioplasty is often required after trauma, but the procedure is not without complications, including wound infection/dehiscence, hematoma, seizure, bone resorption, sunken bone plate, and reoperation [1, 2, 5]. Titanium meshes are widely used for cranioplasty [4]. Still, some patients at high risk and with soft-tissue defect, local tension, radiotherapy, free flap coverage, soft tissue atrophy, infection, and/or chronic rejection will have poor outcomes, including the exposure of the titanium mesh [3, 6, 7, 10, 12, 14, 17]. A study showed that the retention rate of titanium meshes after a median follow-up of 3.9 years was 90% in 177 patients [7]. Children might be at higher risk of mesh exposure because of growth and osteogenesis [14].

A case series suggested that exposed titanium meshes should be removed and the wound repaired with flaps [11], but this strategy means returning to square one for many patients with symptoms due to a non-rigid graft. For selected cases, the exposed titanium mesh can be repaired using the suture of dissociating scalp around the exposed titanium mesh if the exposure area is small, but it is prone to re-exposure over the long term [15]. If the exposed titanium mesh area is large and in an area with extensive infection, the removal of the titanium mesh is the only choice, and then the free flap is used to repair the defect wound, again with disadvantages associated with the loss of the rigid prosthesis.

The present study aimed to report our experience with negative pressure wound therapy combined with soft tissue dilation for retaining the titanium mesh in patients with titanium mesh exposure after cranioplasty.

## Methods and materials

### Patients

This retrospective study included patients with exposed titanium mesh after cranioplasty treated between January 2016 and May 2019 at the Department of Burns and Plastic Surgery of Jiangyin Hospital Affiliated to Southeast University School of Medicine and intending to retain the titanium mesh. The inclusion criteria were (1) 18–80 years of age; (2) exposure of the titanium mesh after cranioplasty to repair a skull defect due to craniocerebral trauma, cerebrovascular disease, intracranial tumor, decompressive craniectomy, etc.; and (3) the exposed area of the titanium mesh was > 1.5 cm^2^, and the thinning area of the surrounding skin was ≥ 5 cm^2^ at admission. The exclusion criteria were (1) systemic diseases (such as autoimmune diseases, hematological diseases, and diabetes) that affected wound healing, (2) a large range of scar tissues around the exposed titanium mesh, or (3) the exposed titanium mesh was combined with local malignant changes.

This study was approved by the Ethics Committee of Jiangyin Hospital Affiliated to Southeast University School of Medicine [ethical approval number: (2019) Lun Shen Yan No. 015].

### Preoperative preparation

**Fig. 1:**
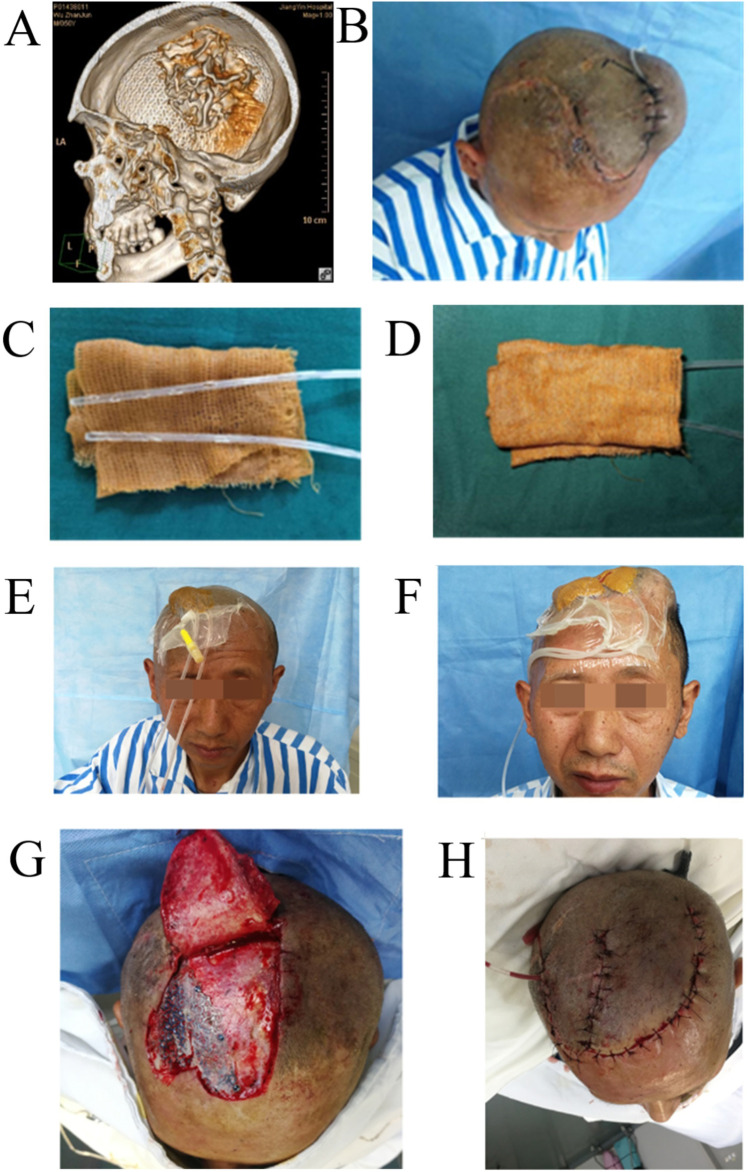
The therapeutic effects of keeping the titanium mesh for exposed titanium mesh after cranioplasty. **A** Computed tomography (CT) 3D reconstruction showing an infection between the titanium mesh and the dura. **B** The skin soft tissue expander was embedded in the loose connective tissue layer beneath the cap aponeurosis 3–6 cm from the exposed edge of the titanium mesh. **C**–**D** Self-made negative pressure wound therapy materials: silver ion dressing wrapped side wall hole disposable sputum suction tube. **E** Negative pressure wound therapy was performed for the exposed titanium mesh. A cannula was inserted between the titanium mesh and the dura mater. **F** Soft tissue expander inserted around the exposed titanium mesh, and negative pressure drainage was continued at the lesion. **G** Scalp transposition after dilation to repair the exposed titanium mesh. **H** An adjacent skin flap was taken to repair the defect at the donor area

Preoperatively, examinations were completed to exclude contraindications to surgery. For those with hypertension, anemia, and hypoproteinemia, adjustment and correction were conducted before surgery. Head computed tomography (CT) and 3D reconstruction were performed to understand the size and location of the titanium mesh, the presence or absence of space between the titanium mesh and the dura mater, and the scope and depth of infection (Fig. 1A). Color Doppler ultrasound of the donor area was performed to understand and mark the direction of the blood vessels and to determine the position of the soft tissue dilator. The peripheral blood inflammation indicators were determined. Bacterial cultures and drug sensitivity tests were conducted. According to the results, vancomycin 1.0 g q 12 h or cefoperazone and sulbactam 2.0 g q 8 h were given. The course of treatment was 5 days. The affected part was rinsed once per day with a debridement instrument (Q/RC-1 type, Urumqi Rongcheng Medical Equipment Co., Ltd., O_3_ water concentration 4–5 mg/l) to clean the wound bed.

### Pretreatment before wound repair

The surgery was performed by a surgeon with > 15 years of experience in burns and plastic surgery. Under general anesthesia, at 3–6 cm from the edge of the exposed titanium mesh, an incision was made vertically or parallelly in the direction of the long axis of the soft tissue dilator to cut the scalp to the subgaleal loose connective tissue, with a vertical incision of 4–5 cm in length and a parallel incision of 5–7 cm in length (Fig. 1B). A cystic cavity was separated under the subgaleal loose connective tissue layer, and the volume of the cystic cavity was slightly larger than that of the dilator. After hemostasis, a soft tissue dilator and disposable suction tube with a lateral opening (Suzhou New District Huasheng Medical Instrument Co., Ltd., tube diameter of 4.0 mm) were placed into the cystic cavity. The injection port was placed outside, and the incision was sutured using an absorbable suture (2/0). The amount of water injected into the dilator during surgery for patients with a vertical incision was 30% of the dilator capacity. Then, a disposable intravenous indwelling tube (Suzhou BD Medical Devices Co., Ltd., specification: 24 G × 0.75 in, 0.7 × 19 mm) was placed into the space between the titanium mesh and the dura mater through the titanium mesh. The drainage tube through the lateral wall opening was wrapped with 2–3 layers of silver ion dressing (Shenzhen Aijiete Medical Technology Co., Ltd., specification: 50 × 40 cm) and placed on the surface of the exposed titanium mesh (hereinafter referred to as negative pressure treatment device, Fig. 1C and D). The transparent film dressing (Smith & Nephew, UK, model: 4631, specification: 20 × 15 cm) was used to close the dilator incision and exposed titanium mesh lesions (Fig. 1E and F). For the above-mentioned drainage tube in the dilated cavity and surface drainage tube of titanium mesh, a T-pipe wall connection medical suction apparatus (model: 882VR-160-1L) and a DL negative pressure waste liquid collection bag (Gentec (Shanghai) Corporation) were used to adjust the negative pressure between − 16.6 and − 10.6 kPa (1 mmHg = 0.133 kPa). Daytime interruption (stopping for 15 min every 2 h of suction) and continuous negative pressure suction mode at night were used. In the interval of negative pressure suction, trypsin (SPH No. 1 Biochemical & Pharmaceutical Co., Ltd.) 50,000 IU and 10 ml of water for injection were dripped into the space between the titanium mesh and the dura mater through the intravenous indwelling tube twice a day for 5 consecutive days. When the expansion of the scalp reached a predetermined value, the repair of the exposed titanium mesh was performed.

### Repair methods of exposed titanium mesh

All patients underwent general anesthesia. Based on the position of the exposed titanium mesh, the patients were placed in the supine or lateral position. The thinned scalp at the edge of the exposed titanium mesh was removed, and the exposed titanium mesh was repeatedly washed with 3% hydrogen peroxide and normal saline. An appropriate auxiliary incision was designed on the dilated scalp, and the scalp flap was transferred to cover the exposed titanium mesh. The distal end of the flap was 4–5 cm beyond the edge of the exposed titanium mesh (Fig. 1G), and it underwent interrupted suture with absorbable suture. If an exposed wound was present in the donor area, the proximal scalp flap was transferred to close the donor area (Fig. 1H). Self-made negative pressure wound therapy was continued for 7 days in the operative area, and then it was changed to routine dressing change until the wound healed.

### Data collection

Sociodemographic characteristics of the patients, preoperative head computed tomography (CT), 3D reconstruction examination, color Doppler ultrasound, and bacterial culture data were collected. Intraoperative and follow-up data were also collected.

## Results

### Characteristics of the patients

During the study period, 11 patients underwent treatments for retaining the titanium mesh after exposure. Two patients were excluded due to autoimmune diseases and one due to diabetes; those three patients did not undergo the procedure described in this study because of uncertain healing outcomes. Therefore, this study finally included eight patients (seven males and one female) (Table 1).

**Table 1:** General data of eight patients with exposed titanium mesh after cranioplasty

#	Sex	Age	Cause of injury	Duration of cranioplasty (days)	Time from exposure of titanium mesh to cranioplasty (days)	Exposed area of titanium mesh (cm^2^)	Thinned area of surrounding skin for exposed titanium mesh (cm^2^)	Space between the titanium mesh and the dura mater (cm)
1	M	47	Trauma	180	90	2.3/3.6	22.0	1.10
2	M	65	Trauma	210	90	3.0	14.0	0.6
3	F	46	Trauma	240	135	4.0	15.6	0.5
4	M	45	Glioma	180	180	3.5	18.6	0.8
5	M	63	Cerebral hemorrhage	270	150	6.0/2.2	17.4	0.0
6	M	48	Trauma	360	210	20.0	20.0	0.9
7	M	51	Trauma	300	186	22.0	15.6	0.0
8	M	64	Trauma	300	120	1.5	21.5	0.0

A preliminary analysis of the causes of titanium mesh exposure showed two patients with infection caused by suture reaction, four with high scalp tension of the local scar, one with infection after scratching, and one idiopathic case. There were three meshes in the forehead, four in the frontoparietal region, and one in the temporal region. The time from cranioplasty to seeking medical attention for the exposure of the titanium mesh was 1–7 months. The exposed area of the mesh ranged from 1 × 1 to 4 × 5.5 cm. Two patients had two sites of exposure. The thinning scalp area around the exposed titanium mesh ranged from 3.6 × 3.8 to 4 × 5.5 cm. In five cases, the titanium mesh was separated by 0.5–1.1 cm from the dura mater. The potential space between the titanium mesh and the dura was not obvious in three cases.

Regarding the bacterial culture of the wound secretions, there were two patients with *Staphylococcus aureus*, one with *Staphylococcus aureus* and *Staphylococcus epidermidis* (sensitive to vancomycin and sodium fusidate), one with *Corynebacterium* (sensitive to cefoperazone-sulbactam), and one with *Pseudomonas aeruginosa* and *Proteus vulgaris* (sensitive to cefoperazone-sulbactam).

### Treatment of the wounds

Table 2 presents the treatment characteristics. There were three patients with peripheral blood neutrophils > 75% and five with C-reactive protein (CRP) > 10 mg/l. After treatment with sensitive antibiotics for 5 days, the wound bacteria turned negative, and CRP and peripheral blood neutrophils decreased within the normal range. A total of 13 soft tissue dilators were used for all patients, including four spherical dilators, with a capacity of 100–200 ml, five kidney-shaped, with a capacity of 100–300 ml, and four rectangular, with a capacity of 250–300 ml. The embedding time was 20–28 days. The time for negative pressure wound therapy was 25–33 days. The hospital stay was 30–41 days.

**Table 2:** Treatment-related data of eight patients with exposed titanium mesh after cranioplasty

#	Embedding time of dilator (days)	Negative pressure treatment time for exposure of titanium mesh (days)	Bacterial culture	Hospital stay (days)	Follow-up time (days)	Prognosis
1	20	25	*Staphylococcus aureus* + *Staphylococcus epidermidis*	41	210	Good
2	28	32		40	180	Good
3	26	30	*Staphylococcus aureus*	36	210	Good
4	28	33	*Staphylococcus aureus*	41	270	Good
5	27	32	–	38	180	Good
6	27	30	*Corynebacterium*	35	260	Good
7	28	32	*Pseudomonas aeruginosa* + *Proteus vulgaris*	40	540	Good
8	28	33	–	40	360	Good

Three months after discharge, the patients were told to return to the hospital for reexamination. Different degrees of hypertrophic scars were present at the distal end of the scalp flap, with poor local mobility, and a certain tension was noted when touched. The head CT scans were unremarkable. After discharge, the conditions were good at each follow-up, and there were no signs of recurrence after 6–18 months of follow-up (Table 2).

## Discussion

The results indicate that negative pressure wound therapy combined with soft tissue dilation for exposed titanium mesh after cranioplasty might help retain the titanium mesh.

A titanium mesh is an optimal material as a skull substitute [3, 4, 6, 7, 10, 12, 14, 17, 20], but the titanium plate or mesh can be gradually exposed after surgery due to factors such as subcutaneous hydrops, hematocele, cerebrospinal fluid leakage, loosening and warping, local high tension state of the scalp caused by a large titanium mesh, too thin separation of scalp flap, the sharp edge of titanium mesh and chronic cutting of scalp, and radiotherapy, among others [3, 6–8, 10, 12, 14, 17–19]. In the present case series, six patients underwent cranioplasty after trauma, one after cerebral hemorrhage, and one after glioma surgery. The causes of mesh exposure were infection caused by suture reaction, high scalp tension of the local scar, and infection after scratching; there was one idiopathic case.

The exposed titanium mesh is generally accompanied by hydrops or even empyema under the titanium mesh. The infected lesion should be removed as soon as possible, and the granulation tissue should quickly fill the space between the titanium mesh and the dura mater [16]. In the present report, a self-designed negative pressure drainage device was used to cover the exposed titanium mesh so that the exposed area of the titanium mesh was under a negative pressure drainage state. An intravenous indwelling tube was placed in the space between the titanium mesh and the dura mater, and 5000 IU/ml trypsin was dripped into the titanium mesh through the indwelling cannula to remove the infection as soon as possible. Given that trypsin is an endopeptidase, it selectively acts on denatured proteins to hydrolyze them into peptides or amino acids to improve tissue permeability, inhibit edema, induce an inflammation response around the thrombus, dissolve blood clots, exudates, necrotic tissues, and decompose viscous secretions [13, 21]. Moreover, it can dissolve the thinned necrotic tissues and be drawn out of the body through the drainage tube, which positively affects cleaning the wound and promotes granulation filling. In order to shorten the treatment cycle, a soft tissue dilator should be placed around the exposed titanium mesh, and a drainage tube should be placed in the dilator implanted cyst, which can effectively prevent intracystic hematoma. On day 3 after surgery, the drainage tube can be removed, and the drainage tube should be wrapped with a silver ion dressing and placed on the incision surface of the dilator. Another drainage tube should be placed on the surface of the titanium mesh through the T-pipe to jointly connect the negative pressure device, which can help prevent incision infection and promote wound healing. The silver ion dressing used to wrap the drainage tube can slowly release silver ions to the wound surface, superior to the VSD wound protection material polyurethane or polyvinyl alcohol in controlling infection [9]. The incision was made perpendicular to the long axis of the soft tissue dilator, which can reduce the tension of the incision to a certain extent to prevent complications such as exposure of the dilator after water injection, and it is beneficial to inject more water during surgery to shorten the course of the disease.

This study has limitations. The sample size was small because of the relative rarity of cranioplasty using a titanium mesh and because the patients were from a single hospital. In addition, due to the study’s retrospective nature, the data that could be analyzed were limited to those available in the charts.

## Conclusion

In conclusion, negative pressure wound therapy combined with soft tissue dilation for exposed titanium mesh after cranioplasty may help retain the titanium mesh. Future studies could look at ways of further improving this technique.

## Data Availability

The data that support the findings of this study are available from the corresponding author upon reasonable request.

## Abbreviations

MRI: Magnetic resonance imaging
CT: Computed tomography
CRP: C-reactive protein
